# The Prognosis of Endometriosis Correlates With Elevated Expression of LncRNA-ANRIL

**DOI:** 10.1155/ogi/9530832

**Published:** 2025-07-26

**Authors:** Gao Jiayin, Quratul Ain, Sun Haizhu, Xiaohong Qiu, Zhang Song

**Affiliations:** ^1^Department of Obstetrics and Gynecology, Second Affiliated Hospital of Harbin Medical University, Harbin, China; ^2^Department of Medical Records, First Affiliated Hospital of Harbin Medical University, Harbin, China

**Keywords:** endometriosis, lncRNA-ANRIL, RT-PCR, Transwell assay

## Abstract

**Background:** Endometriosis is a chronic condition that affects the endometrium, the lining of the uterus. The endometrium typically thickens and discharges during the menstrual cycle, resulting in menstruation. Endometriosis is characterized by developing endometrial-like tissue outside of the uterus, typically on the ovaries, fallopian tubes, and other pelvic structures. This tissue can become inflamed, resulting in various symptoms, such as discomfort. Endometriosis is characterized by heavy menstrual bleeding, fatigue, painful urination or bowel movements, and infertility. Endometriosis is a benign pathological condition frequently seen in the gynecology department. This study classified 28 lncRNAs associated with endometriosis and other gynecological disorders and examined the expression of lncRNA-ANRIL in the eutopic and ectopic endometrium of patients with Ems.

**Methods:** Quantitative reverse transcription (qRT)-PCR was utilized to explore the differences in ANRIL expression between endometriosis tissues and normal ovarian epithelium. Using this technique, the expression of ANRIL in vivo was assessed in 30 endometriosis specimens. A human endometriosis cell line was subjected to in vitro ANRIL knockdown so that the biological roles of the line could be discovered. The Transwell assay was successful in identifying migration and invasion.

**Results:** The expression of ANRIL was much higher in endometriosis tissues than in normal ovarian epithelial tissues, and this difference was found to be strongly associated with the endometriosis stage.

**Conclusions:** There was a positive correlation between the expression of ANRIL and the occurrence of endometriosis. Additionally, there was a close association between the expression of ANRIL and the etiology and development of endometriosis. This offers a potential basis for the early detection and treatment of endometriosis.

## 1. Introduction

Endometriosis is a nonmalignant gynecological condition distinguished by invasive biological characteristics, such as invasion, metastasis, and recurrence [1]. It significantly diminishes patients' quality of life, resulting in symptoms like dysmenorrhea, persistent pelvic discomfort, dyspareunia, menstrual irregularities, and infertility [2]. The increasing incidence of endometriosis highlights the critical need for efficient treatment approaches. Despite being primary treatments, hormone therapy and surgery have poor success because to significant recurrence rates, which may exceed 40% even, postsurgery [1]. These issues underscore the need for an enhanced comprehension of the molecular processes that govern endometriosis to formulate tailored and sustainable therapies.

Long noncoding RNAs (lncRNAs), a type of noncoding transcripts exceeding 200 nucleotides, modulate gene expression at transcriptional, post-transcriptional, and epigenetic levels [3]. Recent evidence indicates that lncRNAs have a role in the pathogenesis of several illnesses, including cancer, by regulating essential cellular processes such as proliferation, apoptosis, and metastasis [3, 4]. The role of lncRNAs in endometriosis remains significantly underexplored, despite their increasing recognition in disease biology. Considering the same molecular pathways linking endometriosis and cancer, including unregulated cell proliferation, tissue invasion, and resistance to apoptosis, lncRNAs provide a potential approach for elucidating the etiology of endometriosis and discovering new therapeutic targets.

Among lncRNAs, Antisense Noncoding RNA in the INK4 Locus (ANRIL) has attracted considerable interest owing to its association with several illnesses, including cancer and cardiovascular conditions [5]. ANRIL, which is situated in the CDKN2A/B gene cluster on chromosome 9p21, regulates gene expression by interacting with polycomb repressive complexes (PRC1 and PRC2), thereby influencing gene methylation and cellular differentiation [6–13]. In cancer, ANRIL promotes tumor growth by repressing tumor suppressor genes and activating oncogenic pathways, including STAT1 and NF-κB [13–17]. These pathways are especially pertinent in endometriosis, where angiogenesis, immunological dysregulation, and chronic inflammation are important factors [18]. For example, the survival and proliferation of endometriotic lesions are facilitated by the activation of NF-κB, whereas STAT1 signaling contributes to inflammatory responses [18, 19]. Moreover, ANRIL's involvement in epithelial-mesenchymal transition (EMT), a critical mechanism for the invasion and formation of endometriotic lesions, highlights its potential significance in endometriosis [20]. EMT allows epithelial cells to transform into mesenchymal cells, which facilitates migration and implantation at ectopic sites. Dysregulation of ANRIL may interfere with EMT pathways, including TGF-β and Wnt/β-catenin, which are inappropriately activated in endometriosis [21].

Nevertheless, the precise function of ANRIL in endometriosis remains unexplored, despite these compelling connections. ANRIL's expression and function in endometriosis tissues and cell lines could offer valuable insights into its role in disease pathogenesis. ANRIL may affect apoptosis resistance, a characteristic of endometriosis, by regulating antiapoptotic genes or engaging with signaling pathways such as PI3K/Akt and MAPK. Comprehending these pathways may uncover new therapeutic targets, especially for individuals with recurring or treatment-resistant conditions [21].

In conclusion, lncRNAs, particularly ANRIL, are expected to significantly influence the molecular pathways associated with endometriosis, including inflammation, EMT, angiogenesis, and apoptosis. The objective of this study is to demonstrate the potential of ANRIL as a diagnostic biomarker and therapeutic target in endometriosis by investigating its expression and functional impact. This will provide new opportunities for the enhancement of the management of this debilitating condition.

## 2. Methods

### 2.1. Patients

Eutopic and ectopic endometrial samples were collected from 30 patients diagnosed with endometriosis at the Second Affiliated Hospital of Harbin Medical University from April 2018 to April 2019. The diagnosis was validated with transvaginal ultrasonography (TVS), pelvic examination, and patient symptom history, then corroborated by histological analysis. The subjects, aged 30–46 years (mean age: 37.80 ± 4.634 years), were premenopausal women with a reproductive history who had not had hormone treatment in the six months before surgery. Of the samples, 14 instances were in the proliferative phase of the endometrium, whereas 16 cases were in the secretory phase. According to the updated American Society of Reproductive Medicine (rASRM) staging guidelines, patients were classified into two groups: 17 with stage I-II endometriosis and 13 with stage III-IV endometriosis.

The control group included 30 persons aged 45–54 years (mean age: 48.53 ± 2.460) who had complete or subtotal hysterectomy for benign diseases, including uterine fibroids or ovarian cysts, with verified nonendometrial pathology. Notably, this research did not contain any cancer patients. The age did not exhibit a significant difference between the endometriosis and control groups (*p* > 0.05). The exclusion criteria were pregnancy, breastfeeding, intrauterine device (IUD) use, and any prior hormone therapy within 6 months before surgery, since hormonal treatments are often employed to alleviate endometriosis symptoms and may distort the findings.

The quality and representativeness of the tissue samples were ensured by meticulous histological verification in order to address the issues related to ovarian endometriosis lesions, such as the paucity of endometriosis cells as a result of the toxic microenvironment of ovarian endometrioma. Eutopic endometrial tissues from the same patients served as controls to reduce variability and for a more precise comparison, given the contentious nature of using ovarian surface epithelium owing to persistent discussions on the cellular genesis of endometriosis. This methodology improves the study's validity by guaranteeing that the control tissues are physiologically relevant and equivalent to the ectopic endometrial specimens.

This research attempts to minimize any confounding variables and offer a strong and representative examination of the molecular pathways driving endometriosis by using eutopic endometrium from the same patients as controls and omitting cancer patients.

### 2.2. Selection and Analysis of LncRNAs

This study selected 28 lncRNAs based on their established associations with endometriosis and other benign and malignant gynecological conditions, as documented in the literature. The PANTHER Classification System (https://www.pantherdb.org/) was utilized to characterize the molecular functions, biological processes, and related protein functionalities of these lncRNAs. This bioinformatics tool enabled a systematic analysis of the selected lncRNAs, offering insights into their potential roles in the pathogenesis of endometriosis.

### 2.3. Real-Time Polymerase Chain Reaction (RT-PCR)

Total RNA was isolated from tissue samples and cell lines with TRIzol reagent (Invitrogen, CA, USA). The purity of RNA was evaluated by determining the optical density (OD) ratio at 260/280 nm, with ratios ranging from 1.8 to 2.0 being acceptable. Reverse transcription was conducted with oligo(dT) primers and a reverse transcriptase kit (Bioneer, Shanghai, China) to generate complementary DNA (cDNA) from 1 μg of total RNA. The 20 μL reaction mixture was incubated in a PCR system at 56°C for 1 min, 50°C for 60 min, and 95°C for 5 min, then maintained at 4°C.

Quantitative RT-PCR (qRT-PCR) was performed using the ABI 7300 RT-PCR machine (Applied Biosystems, CA, USA). The reaction conditions included an initial denaturation phase at 95°C for 5 min, followed by 40 cycles of 95°C for 30 s and 55°C for 30 s. The primer sequences used in this investigation were constructed using Primer3 software (Shanghai Generay Biotech Co. Ltd.) and are as follows:

  LncRNA-ANRIL-F: TCTCACAGCCTGGAAGGAGT
  LncRNA-ANRIL-R: GCAGACACTGCATGGAAGAA
  β-actin-F: TGAAGTGTGACGTGGACATC
  β-actin-R: GGAGGAGCAATGATCTTGAT

All qRT-PCR reactions were conducted in triplicate, and the threshold cycle (CT) values were ascertained using automatic settings. The relative expression of ANRIL was determined using the 2-ΔΔCt technique, normalized to the endogenous control β-actin.

### 2.4. Cell Culture and Transfection

The human endometriosis cell lines used in this study were obtained from eutopic and ectopic endometrial tissues of a 41 year-old female patient diagnosed with endometriosis. The cell lines' validity was validated by Short Tandem Repeat (STR) profiling, and mycoplasma contamination was ruled out using a mycoplasma detection kit. These cell lines were selected for their significance to endometriosis, since they preserve the molecular and phenotypic traits of endometrial cells, including hormone responsiveness and invasive capability.

Endometrial cells were extracted from fresh tissue specimens and grown in Dulbecco's Modified Eagle Medium (DMEM) enriched with 10% fetal bovine serum (FBS) and 1% streptomycin. Cells were sustained in a humidified incubator at 37°C with 5% CO_2_ concentration. For transfection studies, cells in the logarithmic growth phase were trypsinized, enumerated, and plated onto 6-well plates at a density of 5 × 10 ^ 5 cells per well. When cells attained 50%–60% confluency, they were transfected with small interfering RNAs (siRNAs) directed against ANRIL (si-ANRIL) or a negative control siRNA (si-NC) using Lipofectamine 2000 reagent (Invitrogen, CA, USA) in accordance with the manufacturer's instructions. Cells that were transfected were collected 48 h after transfection for further functional tests.

### 2.5. Transwell Assay

Cell migration and invasion Assays were conducted using Costar Transwell chambers (Corning Costar Corp., Cambridge, MA, USA). For the migration test, 5 × 10 ^ 4 transfected cells were suspended in serum-free media and placed in the top chamber of an uncoated Transwell insert with an 8-μm pore size. The top chamber was precoated with Matrigel (BD Biosciences, San Jose, CA, USA) for the invasion experiment to replicate the extracellular matrix. The bottom compartment was populated with medium containing 10% FBS as a chemoattractant. Following 48 h of incubation at 37°C, nonmigrated or noninvaded cells on the top membrane surface were meticulously excised using a cotton swab. Cells that migrated or colonized the lower surface were fixed with 4% paraformaldehyde and stained with 0.5% crystal violet. The cell count was determined by analyzing five random fields per membrane using Image-Pro Plus 6.0 software (Media Cybernetics, Rockville, MD, USA). Every experiment was conducted in triplicate to guarantee repeatability.

### 2.6. Statistical Analysis

Statistical analysis was conducted using SPSS 17.0 (SPSS, Inc., Chicago, IL, USA). The data are presented as means ± standard error of the mean from a minimum of three independent experiments. Statistical analysis included Chi-square and Student's *t*-tests and one-way analysis of variance followed by a post hoc test when applicable. Statistical significance was considered at a *p* value of less than 0.05 (*p* < 0.05).

## 3. Results

### 3.1. Biological Analysis of 28 Known LncRNAs

**Figure 1:**
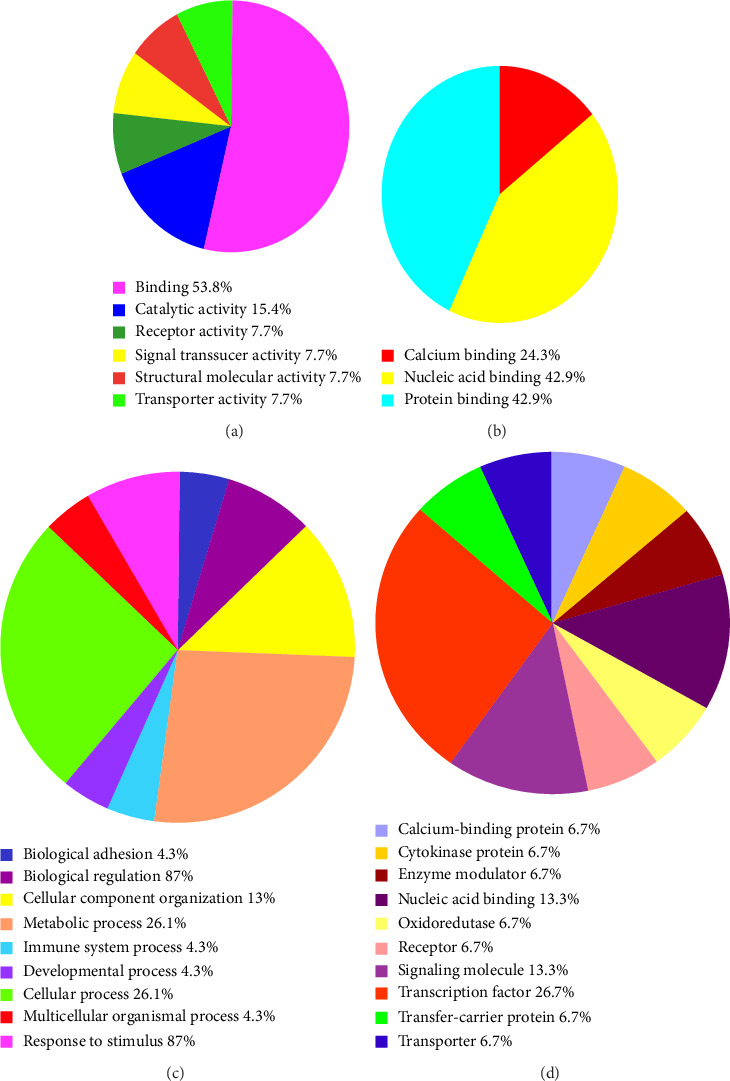
Analysis of biological characteristics of 28 lncRNAs. (a) Molecular function. (b) Binding function. (c) Biological process. (d) Protein class.

This research categorized the molecular functions of lncRNAs into specific classifications, as illustrated in Figure 1(a). The lncRNAs exhibited a range of functional roles, including biological binding, protein binding, signal transduction, receptor activation, regulation of molecular structures, and activation of molecular transporters. The primary function identified was biological binding, accounting for 53.8% of the recorded activities (Figure 1(b)). This category examines interactions between proteins and nucleic acids, highlighting the critical role of lncRNAs in facilitating molecular interactions that regulate cellular processes.

The lncRNAs demonstrated notable correlations with several essential biological processes, as shown in Figure 1(c). Cell cycle regulation and substance metabolism were identified as the primary processes, each comprising 26.1% of the overall functional profile. lncRNAs are linked to critical processes such as immune response, biological regulation, and adhesion, underscoring their varied roles in maintaining cellular homeostasis and responding to environmental stimuli.

The classification of proteins associated with these lncRNAs revealed that transcription factors represented the largest category, constituting 26.7% of the total (Figure 1(d)). Nucleic acid-binding proteins and signaling molecules subsequently emerged, underscoring the regulatory role of lncRNAs in gene expression and cellular signaling pathways. The relationship between lncRNAs and transcription factors, as well as nucleic acid-binding proteins, underscores their critical role in the regulation of transcriptional networks and epigenetic processes. Their interaction with signaling molecules highlights their function in intracellular and intercellular communication.

### 3.2. Expression of LncRNA-ANRIL in Clinical Samples

**Figure 2:**
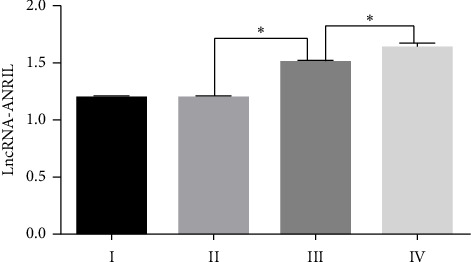
Expression of lncRNA-ANRIL in endometrium of different stages of ASRM. There was no significant difference in the expression of lncRNA-ANRIL between stage I and stage II ectopic lesions, but the expression in the ectopic lesions increased with stage from stage II to IV (*p* < 0.05).

**Figure 3:**
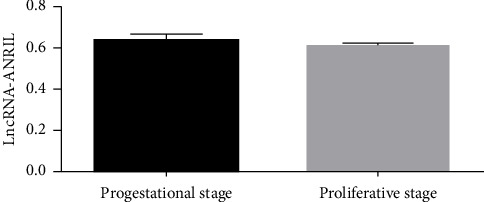
Expression of lncRNA-ANRIL in different phases of endometrium. There was no significant difference in the expression of lncRNA-ANRIL between the secretory phase and proliferative phase of endometrium (*p* > 0.05).

**Figure 4:**
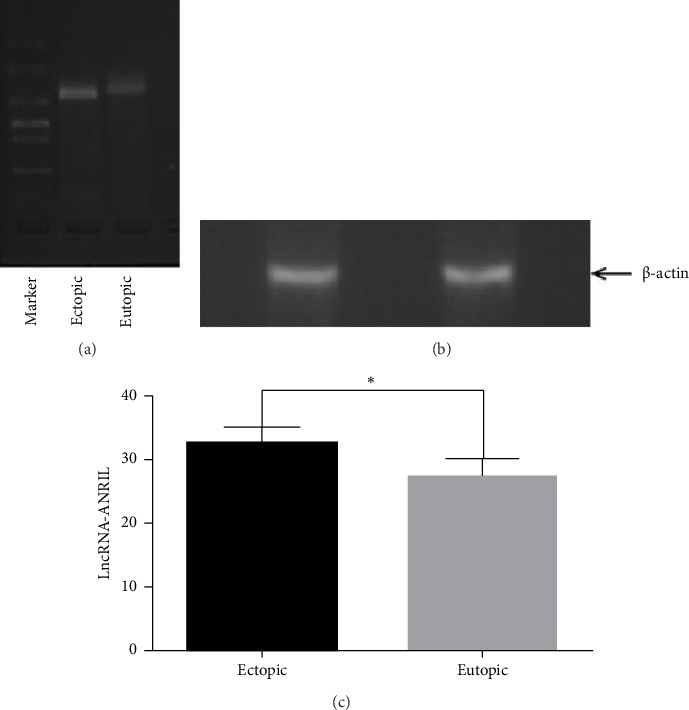
Expression of lncRNA-ANRIL in ectopic and eutopic endometrium. (a) Electrophoresis of RT-PCR products showed that the expression of lncRNA-ANRIL in ectopic tissue was higher than that in eutopic endometrium (*p* < 0.05). (b) Expression of β-actin in ectopic and eutopic endometrium of uterus showed no statistically significant difference in the amount of total RNA for the two groups (*p* > 0.05). (c) Bar graph showing the expression of lncRNA-ANRIL (*p* < 0.05).

**Figure 5:**
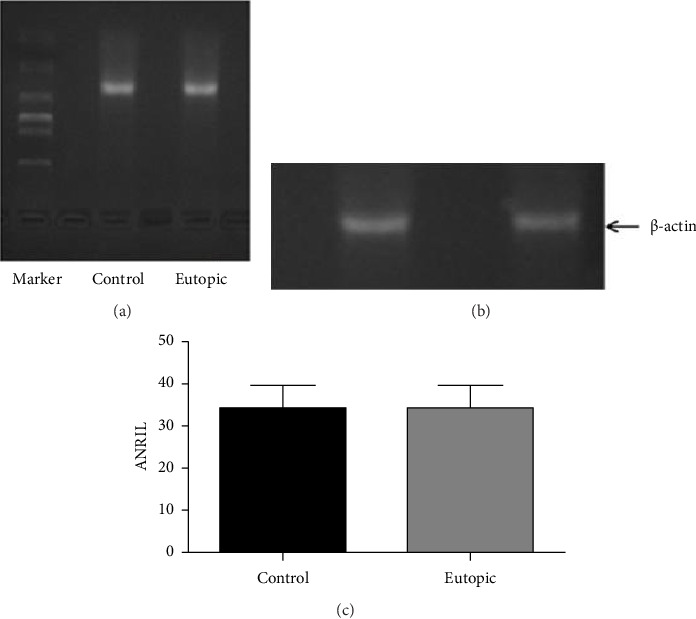
Expression of lncRNA-ANRIL in control and ectopic endometrium. (a) Electrophoresis of RT-PCR products showed no significant difference in the expression of lncRNA-ANRIL between the control group and ectopic endometrium (*p* > 0.05). (b) There was no statistical difference in the expression of β-actin in ectopic and eutopic endometrium (*p* > 0.05). (c) Bar graph showing the expression of lncRNA-ANRIL (*p* < 0.05). There was no difference in the expression of lncRNA-ANRIL in ectopic endometrium between the control group and the endometriosis patients.

The expression of lncRNA ANRIL was evaluated in eutopic and ectopic endometrial tissues from patients with endometriosis through RT-PCR. Gel electrophoresis on a 2% agarose gel revealed a distinct single band corresponding to a PCR product of approximately 400 base pairs, thereby confirming the specificity of the primers and the accurate identification of lncRNA ANRIL in all tissue samples. Gel images were quantified with ImageJ software, and a paired *t*-test was performed in SPSS 17 to assess the expression patterns of lncRNA ANRIL in relation to various clinical factors. The results indicated no significant differences in lncRNA ANRIL expression among different age groups (*p* > 0.05), implying that aging does not influence its expression. No significant difference in lncRNA ANRIL expression was observed between patients with stage I and stage II endometriosis (*p* > 0.05). A significant rise in lncRNA ANRIL expression was observed as illness severity advanced from stage II to stage IV (*p* < 0.05), as detailed in Table 1 and illustrated in Figure 2. This discovery suggests that lncRNA ANRIL may play a crucial role in the progression of endometriosis, particularly in its advanced stages. The expression of lncRNA ANRIL demonstrated no significant association with the phases of the menstrual cycle, as no variations were observed between the secretory and proliferative phases of the endometrium (*p* > 0.05), as illustrated in Figure 3 and summarized in Table 2. These findings indicate that hormonal fluctuations associated with the menstrual cycle do not significantly influence lncRNA ANRIL expression. The expression of lncRNA ANRIL was significantly increased in ectopic lesions compared to eutopic endometrium (*p* < 0.05), as illustrated in Figure 4, suggesting its potential involvement in the pathogenic mechanisms related to ectopic tissue survival, invasion, and disease progression. No significant difference in lncRNA ANRIL expression was observed between the eutopic endometrium of endometriosis patients and the control endometrium from individuals with benign conditions, such as uterine fibroids or ovarian cysts (*p* > 0.05), as illustrated in Figure 5. This highlights the specificity of lncRNA ANRIL overexpression in ectopic lesions and indicates its potential as a biomarker for the behavior of ectopic endometrial tissue. The data suggest that lncRNA ANRIL may contribute to the development and severity of endometriosis, particularly in advanced stages, and establish a foundation for further investigation into its mechanistic role in disease pathogenesis. The stratification of results by disease stage, as shown in Table 1, offers critical insights into the differing expression patterns of lncRNA ANRIL across various levels of endometriosis severity, underscoring its potential as a therapeutic target or diagnostic marker for advanced disease. Further investigation is required to elucidate the precise molecular mechanisms through which lncRNA ANRIL influences the development of endometriosis and to explore its viability as a novel therapeutic target.

### 3.3. Knockdown of ANRIL Functions in Endometrium Cell Invasion

**Figure 6:**
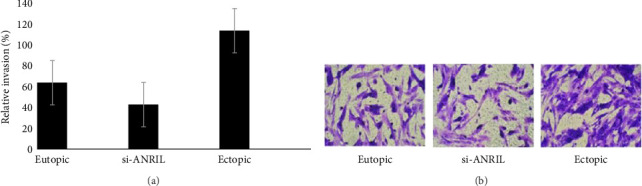
Knockdown of ANRIL functions in endometrium cell invasion. Endometrium cells were transfected with the expression vectors of sh-ANRIL to knockdown ANRIL expression (a). Transwell assay was performed to analyze cell invasion in these transfected cells (*p* < 0.05) (b).

The functional role of ANRIL in cell invasion was examined through a Transwell assay to evaluate the invasive capabilities of endometrial cells after ANRIL knockdown. Endometrial cells underwent transfection with short hairpin RNA (shRNA) directed against ANRIL (sh-ANRIL) to achieve a significant reduction in its expression. The findings indicated that the knockdown of ANRIL markedly reduced the invasive capacity of endometrial cells relative to control groups (*p* < 0.05), as shown in Figures 6(a), 6(b). This finding indicates that ANRIL is essential in facilitating cell invasion, a significant pathological characteristic of endometriosis. It is crucial to acknowledge that the study did not stratify results by disease stage, which is vital for understanding the functional significance of ANRIL in various stages of endometriosis. Stratifying by stage would yield insights into the extent of ANRIL's role in invasion, particularly in advanced stages (III and IV) versus earlier stages (I and II), thus increasing the clinical relevance of these findings.

## 4. Discussion

Endometriosis, while nonfatal, significantly impacts the quality of life and poses challenges due to its malignant biological traits such as invasion, metastasis, and recurrence [1]. Despite its prevalence, the pathophysiology of endometriosis remains incompletely understood, with theories such as Sampson blood circulation planting and Lang Jinghe's “eutopic endometrium determinism” proposed. A deeper understanding of its pathogenesis is crucial for improving treatment outcomes and addressing recurrence issues [16].

LncRNAs have been identified as significant contributors to the pathological progression of endometriosis, providing a new and promising area of study. Wang, Li et al. discovered that there is an abnormal expression of many lncRNAs in both standard and abnormal endometrial tissue [17]. This suggests that these lncRNAs play a role in regulating the cell cycle, which is essential in the progression of endometriosis. In particular, it was proposed that AC002454.1 controls CDK6, a cell cycle regulator, which may impact the pathophysiology of endometriosis [18].

Previous studies have emphasised the significance of lncRNAs in the pathogenesis of several diseases, particularly in the processes of chromosomal remodeling, control of gene expression, and disease advancement [17, 19]. However, their involvement in gynecological disorders remains inadequately comprehended.

Hudson et al. have also investigated the importance of lncRNAs in endometriosis, highlighting their potential involvement in the disease's pathogenesis. Their research provides evidence in favor of the idea that lncRNAs could be used as biomarkers for endometriosis and perhaps informs therapeutic approaches [19].

This study examines the expression and functional role of lncRNA ANRIL in endometriosis, offering insights into its potential involvement in disease progression.

The biological analysis of 28 known lncRNAs demonstrated their diverse functional roles, encompassing biological binding, protein binding, signal transduction, receptor activation, and regulation of molecular structures and transporters. Biological binding, mainly concerning interactions with proteins and nucleic acids, constituted the predominant function, representing 53.8% of the recorded activities (Figure 1(b)). This underscores the essential function of lncRNAs in facilitating molecular interactions that govern cellular processes. Additionally, lncRNAs exhibited a significant correlation with critical biological processes, including cell cycle regulation and substance metabolism, each accounting for 26.1% of the overall functional profile (Figure 1(c)). The findings highlight the diverse functions of lncRNAs in preserving cellular homeostasis and reacting to environmental stimuli. The classification of proteins linked to these lncRNAs indicated that transcription factors represented the largest category at 26.7%, followed by nucleic acid-binding proteins and signaling molecules (Figure 1(d)). This indicates that lncRNAs are crucial in transcriptional regulation, epigenetic modifications, and cellular signaling pathways, highlighting their potential as significant regulators in disease pathogenesis.

Our evaluation of lncRNA ANRIL expression in clinical samples revealed no significant differences among age groups (*p* > 0.05), suggesting that age does not affect its expression in endometriosis. No significant difference in lncRNA ANRIL expression was observed between patients with stage I and stage II endometriosis (*p* > 0.05). A notable increase in lncRNA ANRIL expression was detected as the disease progressed from stage II to stage IV (*p* < 0.05), as presented in Table 1 and depicted in Figure 2. This indicates that lncRNA ANRIL may be significant in the advanced stages of endometriosis, possibly influencing the aggressive characteristics of ectopic lesions. lncRNA ANRIL expression showed no correlation with the menstrual cycle phase (secretory vs. proliferative, *p* > 0.05), as illustrated in Figure 3 and summarized in Table 2, suggesting that hormonal fluctuations do not significantly affect its expression. lncRNA ANRIL expression was significantly elevated in ectopic lesions relative to eutopic endometrium (*p* < 0.05), as illustrated in Figure 4, indicating its possible role in the pathological mechanisms associated with ectopic tissue survival and invasion. No significant difference was observed between eutopic endometrium from endometriosis patients and control endometrium from individuals with benign conditions (*p* > 0.05), as shown in Figure 5, underscoring the specificity of lncRNA ANRIL upregulation to ectopic lesions.

We performed Transwell assays to investigate the functional role of lncRNA ANRIL in cell invasion. The knockdown of ANRIL via shRNA markedly reduced the invasive ability of endometrial cells (*p* < 0.05), as illustrated in Figures 6(a), 6(b). This finding demonstrates that lncRNA ANRIL is crucial in facilitating cell invasion, a defining characteristic of endometriosis. It is noteworthy that the study did not stratify results by disease stage, which is crucial for comprehending the functional relevance of ANRIL in various stages of endometriosis. Stratifying by stage would yield greater insights into the extent of ANRIL's role in invasion, particularly in advanced stages (III and IV) versus earlier stages (I and II), thus increasing the clinical significance of these findings.

Our findings indicate a potential correlation between lncRNA ANRIL expression and endometriosis progression; however, the limitations of this study must be acknowledged. The limited sample size, absence of validation in independent cohorts, and lack of appropriate controls for specific experiments may restrict the generalizability of the findings. The mechanisms by which ANRIL affects the progression of endometriosis are not fully elucidated. Additional research is required to clarify the specific molecular pathways involved and to confirm ANRIL as a potential biomarker or therapeutic target. The implications of ANRIL in endometriosis require careful consideration, highlighting the necessity for larger, rigorously designed studies to validate these findings.

In summary, this study offers important insights into the expression patterns and functional roles of lncRNA ANRIL in endometriosis. The upregulation of ANRIL in advanced stages and ectopic lesions, coupled with its role in facilitating cell invasion, indicates its potential involvement in the pathogenesis and progression of endometriosis. Nonetheless, the conclusive determination that ANRIL expression is associated with the incidence, causes, and progression of endometriosis necessitates additional validation. Future research should aim to clarify the molecular mechanisms of ANRIL, investigate its roles at different stages, and evaluate its potential as a diagnostic biomarker or therapeutic target. These initiatives may facilitate novel strategies for the management of this intricate and debilitating gynecological condition.

## Figures and Tables

**Table 1:** Expression of lncRNA-ANRIL in different stages of ASRM.

	ASRM			
	I	II	III	IV
*N*	7	10	6	7
$\bar{x}$ ± *s*	1.190 ± 0.003	1.195 ± 0.004	1.502 ± 0.038	1.630 ± 0.018

**Table 2:** Expression of lncRNA-ANRIL in secretory endometrium and proliferative endometrium.

	*N*	$\bar{x}$ ± *s*	*p*
Secretory	16	0.637 ± 0.018	*p* > 0.05
Proliferative	14	0.610 ± 0.010	

## Data Availability

There is no publicly available data for this research. All data were collected and analyzed in accordance with ethical guidelines.
